# Co-encapsulated astaxanthin and kaempferol nanoparticles: fabrication, characterization, and their potential synergistic effects on treating non-alcoholic fatty liver disease†

**DOI:** 10.1039/d3ra06537e

**Published:** 2023-12-01

**Authors:** Ho Thi Oanh, Ngo Thi Hoai Thu, Nguyen Van Hanh, Mai Ha Hoang, Hoang Thi Minh Hien

**Affiliations:** a Institute of Chemistry, Vietnam Academy of Science and Technology 18 Hoang Quoc Viet Street, Cau Giay 10072 Hanoi Vietnam hoangmaiha@ich.vast.vn; b Institute of Biotechnology, Vietnam Academy of Science and Technology 18 Hoang Quoc Viet Street, Cau Giay 10072 Hanoi Vietnam hmhien@ibt.ac.vn; c Graduate University of Science and Technology, Vietnam Academy of Science and Technology 18 Hoang Quoc Viet Street, Cau Giay 10072 Hanoi Vietnam

## Abstract

Astaxanthin and kaempferol, renowned natural compounds, possess potent antioxidant properties and exhibit remarkable biological activities. However, their poor water solubility, low stability, and limited bioavailability are the primary bottlenecks that restrict their utilization in pharmaceuticals and functional foods. To overcome these drawbacks, this study aims to fabricate astaxanthin/kaempferol co-encapsulated nanoparticles and investigate their synergistic effects on reducing the risk of stress oxidation, chronic inflammation, and lipid accumulation in RAW264.7 and HepG2 cells. The synthesized astaxanthin/kaempferol nanoparticles exhibited well-defined spherical morphology with an average particle diameter ranging from 74 to 120 nm. These nanoparticles demonstrated excellent stability with the remaining astaxanthin content ranging from 82.5% to 92.1% after 6 months of storage at 4 °C. Nanoastaxanthin/kaempferol displayed high dispersibility and stability in aqueous solutions, resulting in a significant enhancement of their bioactivity. *In vitro* assessments on cell lines revealed that nanoastaxanthin/kaempferol enhanced the inhibition of H_2_O_2_-induced oxidative stress in HepG2 and LPS-induced NO production in RAW264.7 compared to nanoastaxanthin. Additionally, these nanoparticles reduced the expression of genes involved in inflammation (iNOS, IL-6 and TNF-α). Moreover, hepatocytes treated with nanoastaxanthin/kaempferol showed a reduction in lipid content compared to those treated with nanoastaxanthin, through enhanced regulation of lipid metabolism-related genes. Overall, these findings suggest that the successful fabrication of co-encapsulated nanoparticles containing astaxanthin and kaempferol holds promising therapeutic potential in the treatment of non-alcoholic fatty liver disease.

## Abstract 2

Astaxanthin/kaempferol co-encapsulated nanoparticles were fabricated using cremophor RH40 and lecithin as surface-active agents, and β-cyclodextrin as an encapsulating agent. The nanoparticles showed high dispersibility and stability in aqueous solutions, which enhances their bioactivity.

## Graphical abstract

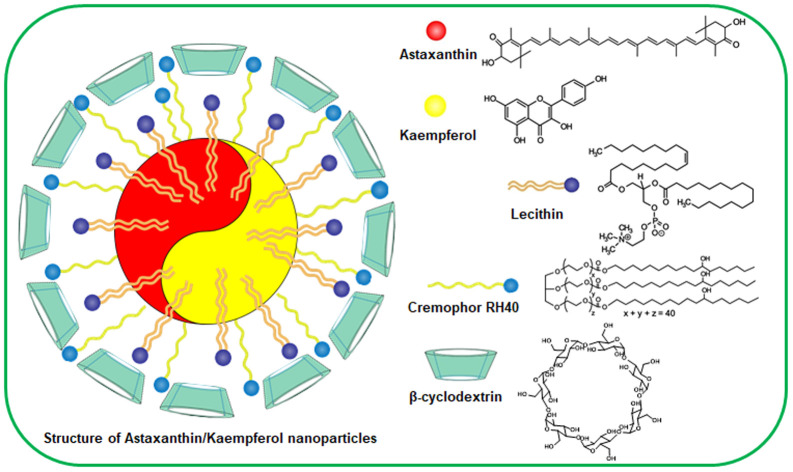

## 1 Introduction

The beneficial impacts of carotenoids and flavonoids like astaxanthin and kaempferol on human health have attracted significant research attention. Astaxanthin, a natural carotenoid pigment, can be synthesized by a number of microorganisms, especially green microalgae *Haematococcus pluvialis* (4–6% of dry biomass).^1^ Compared with other carotenoids, astaxanthin has been shown to exhibit a stronger antioxidant activity and with its properties, AST may potentially help to prevent non-alcoholic fatty liver disease (NAFLD) and atherosclerosis through improving antioxidant defense mechanisms and alleviating dyslipidemia.^2^ A study on high-fat-fed (HFD) mice showed that oral administration of astaxanthin significantly reduced plasma and liver total cholesterol and triglyceride levels compared with the control HFD mice.^3^ In addition, no abnormalities or adverse effects were found in rats given oral supplementation of astaxanthin at a dose of 500 mg kg^−1^ per day; while the LD_50_ of astaxanthin was greater than 20 g per kg bw in pregnant mice administrated astaxanthin at doses of 100, 250 and 500 mg per kg bw.^4^ Moreover, excess intake of astaxanthin will not lead to hypervitaminosis A toxicity because it cannot be converted to vitamin A or synthesized in humans.^5^

Kaempferol is a natural flavonoid that is present in different plant species, seaweed, and common medicinal plants.^6^ Previous studies have reported that kaempferol could prevent the development of NAFLD by targeting multiple targets such as suppressing oxidation, reducing inflammation, hyperlipidemia, and the risk of chronic diseases (obesity, diabetes, liver injury, and cancer) and improving insulin resistance and reducing.^7,8^ Liu *et al.*^9^ showed that kaempferol could inhibit inflammatory responses and prevent the development of NAFLD *in vitro* and *in vivo* by suppressing the nuclear transcriptional activity of nuclear factor-κB (NF-κB). Several epidemiological studies have indicated that the consumption of kaempferol rich foods can reduce the risk of disorders as inflammatory and cardiovascular diseases.^10^ A study on the association between flavonoid-containing foods intake (*i.e.* apples, berries, grapefruit, oranges, juices, white cabbage, and onions) and the risk of several chronic diseases in 10 054 people by a prospective cohort study showed that the incidence of cerebrovascular disease was lower at higher kaempferol intakes (RR = 0.70; 95% CI: 0.56–0.86; p: 0.003; median intake was 0.5 mg kaempferol per day).^11^ Moreover, the amount of kaempferol in the human dietary intake can be estimated at up to approximately 10 mg per day.^12^

Several studies have indicated that different antioxidants are localized in various cellular positions within living organisms. Hence, in many cases, the combination of natural compounds is evaluated for higher antioxidative effects compared to using each individual compound separately.^13,14^ Kawamura *et al.*^15^ reported that the combination of astaxanthin, β-carotene, and resveratrol, even in small amounts, enhanced the protein synthesis process during muscle hypertrophy after atrophy. On the other hand, Oanh and co-workers^16^ also demonstrated that the combination of lycopene and resveratrol enhanced the stability of lycopene. Astaxanthin and kaempferol are strong antioxidants and potent NAFLD therapeutic agents; however, their pharmaceutical uses are limited due to many problems associated with low solubility, permeability, and bioavailability. Moreover, in the case of astaxanthin, its molecular structure contains conjugated double bonds that the compound unstable, leading to its vulnerability to degradation caused by oxidative agents, temperature, and light. On a separate note, with regard to kaempferol, extensive doses of kaempferol have been found to induce toxic effects in both experimental and clinical studies.^6^ In recent years, nanotechnology has opened up promising avenues to enhance the applicability and efficiency of natural bioactive compounds in the fields of pharmaceuticals, food, cosmetics, and medicine. The co-encapsulation of bioactive compounds within nanoparticles has garnered attention as an effective solution for enhancing their bioavailability and synergistic effects.

In this work, we fabricated nanoparticles that co-encapsulated astaxanthin and kaempferol in varying weight ratios to enhance the solubility, stability, and bioactivity of these compounds. For the synthesis of nanoencapsulation, cremophor RH40 and lecithin were chosen as surface-active agents, vitamin E as an antioxidant, and β-cyclodextrin as the encapsulating agent. Subsequently, the nanopowder was formed using the spray drying method. After formation, the nanoparticle characteristics and their stability under various storage conditions were evaluated. Through formula optimization, the astaxanthin/kaempferol nanoparticles were assessed for their efficacy in reducing the risk of oxidative stress, chronic inflammation, and lipid accumulation in RAW264.7 and HepG2 cell lines.

## 2 Experimental

### 2.1. Materials

Astaxanthin was extracted with purity above 96% from the green microalga *Haematococcus pluvialis*.^17^ Kaempferol (95% purity) and lecithin were obtained from AK Scientific, USA. Cremophor RH40, vitamin E, and β-cyclodextrin (purity ≥97%) were purchased from Sigma-Aldrich. 2,2-Diphenyl-1-picrylhydrazyl (DPPH) and 3-(4,5-dimethylthiazol-2-yl)-2,5-diphenyltetrazolium bromide (MTT) were bought from TCI-EP Chemical Co, Tokyo, Japan and Thermo Fisher Scientific, Waltham, USA, respectively. All solvents utilized were of high purity and were employed without additional purification.

### 2.2. Preparation of astaxanthin/kaempferol co-encapsulated nanoparticles

Astaxanthin/kaempferol co-encapsulated nanoparticles with varying amounts of astaxanthin and kaempferol were fabricated using emulsion/solvent evaporation techniques,^18,19^ followed by the obtaining of nanopowder through the spray drying method. This is the most commonly employed methods for the preparation of nanoparticles of bioactive compounds by adjusting the surfactants and encapsulation agents. Briefly, astaxanthin and kaempferol, along with lecithin, cremophor RH40, and vitamin E (Table 1), were completely dissolved in 350 mL of tetrahydrofuran using a mechanical stirrer (RW20 IKA, Malaysia) at 1000 rpm for 45 minutes. Subsequently, the organic phase was slowly added drop by drop into 1 L of deionized water containing β-cyclodextrin. This process was carried out using an Ultra-Turrax homogenizer (T18 IKA, Germany) at 5000 rpm for 60 minutes. Afterward, the astaxanthin/kaempferol nanosuspension was introduced into a spray dryer (B-290, Buchi, Switzerland) with a flow rate set at approximately 1 L h^−1^ and an airflow rate of 32 m^3^ h^−1^. The inlet temperature was set at 115 °C, and the air pressure was set at 6 bar. The nano powder was collected from the particle chamber using a powder scraper and subsequently placed in airtight brown glass bottles for further analysis.

**Table 1:** Composition of astaxanthin/kaempferol co-encapsulated nanoparticles samples

Samples	Astaxanthin (g)	Kaempferol (g)	Cremophor RH40 (g)	Lecithin (g)	Vitamin E (g)	β-Cyclodextrin (g)	Astaxanthin content (%)	Kaempferol content (%)
AK1	2.0	0	4	2	0.4	11.6	10	0
AK2	1.8	0.2	4	2	0.4	11.6	9.0	1.0
AK3	1.5	0.5	4	2	0.4	11.6	7.5	2.5
AK4	1.0	1.0	4	2	0.4	11.6	5.0	5.0
AK5	0.5	1.5	4	2	0.4	11.6	2.5	7.5

### 2.3. Characterization and morphology of astaxanthin/kaempferol nanoparticles

The zeta potential, polydispersity index (PDI), and particle size of the astaxanthin/kaempferol nanoparticles were measured using dynamic light scattering on a Litesizer™ 500 instrument (Anton Paar, Austria). The nanoparticles were evaluated by dispersing them into the deionized water at a concentration of 100 ppm. The measurements were conducted three times, and the results were expressed as the mean value accompanied by the standard deviation of the recorded data.

The morphology of the astaxanthin/kaempferol nanoparticles was determined by using transmission electron microscopy (TEM, JEM 2100, JEOL, Japan). The samples were observed directly without any staining.

### 2.4. Stability of astaxanthin/kaempferol nanoparticles

The stability of the astaxanthin nanopowder was assessed under various storage conditions, including 4 °C and room temperature.

Degradation of astaxanthin within the nanopowder was quantified employing a UV-visible spectrophotometer (Optima SP-3000 nano, Japan). The remaining astaxanthin content was quantified using the subsequent equation:1
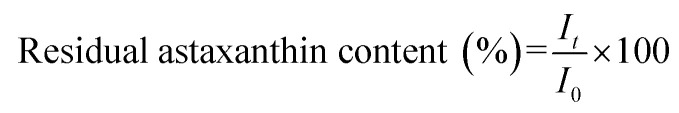
where *I*_0_ is the initial absorbance intensity at 474 nm and *I*_*t*_ is the absorbance intensity at 474 nm after storage time *t*.

### 2.5. DPPH assay

The antioxidant capacity of nanoastaxanthin/kaempferol was determined using the DPPH assay.^17^ In summary, 100 μL of a 0.2 mM DPPH solution in methanol was mixed with 100 μL of either nanoastaxanthin (AK1; 10 wt% AST) or nanoastaxanthin/kaempferol (AK3; 7.5 wt% AST and 2.5 wt% KAE) at varying doses (10–500 μg mL^−1^). Ascorbic acid was used as a positive control. The reaction mixture was incubated at room temperature in the dark for 30 minutes. After 30 minutes, the absorbance of the reaction solutions was measured using a microplate reader (Thermo Fisher Scientific, Inc., Waltham, MA, USA) at 517 nm.

Each experiment was conducted in triplicate, and the results were expressed as IC_50_ values.

### 2.6. Cell culture and treatment

The RAW264.7 (ATTC, TIB-71™) and HepG2 (ATTC, HB-8065™) cell lines were obtained from the American Type Culture Collection (Manassas, VA, USA). The cells were cultured in Dulbecco's Minimum Essential Medium (DMEM) with high glucose concentration (4500 mg L^−1^) and supplemented with 10% fetal bovine serum (FBS), 100 U per mL penicillin, and 0.1 mg per mL streptomycin. The culturing process was conducted in a sterile incubator at 37 °C with 5% CO_2_.

To assess cell viability, RAW264.7 or HepG2 cells were seeded at a density of 0.5 × 10^5^ cells per well in DMEM/high glucose in 96-well culture plates. After 24 hours of cultivation, the cells were further incubated with nanoastaxanthin (AK1; 10 wt% AST) or nanoastaxanthin/kaempferol (AK3; 7.5 wt% AST and 2.5 wt% KAE) at specific concentrations for an additional 24 hours. Cell viability was then analyzed using the MTT assay.

To assess anti-inflammatory activity, RAW264.7 cells were cultured into 6-well culture plates in DMEM/high glucose at a density of 1 × 10^6^ cells per well and cultured 24 hours. Afterward, the cells were treated with 100 μg mL^−1^ of AK1 or AK3 for 2 h followed lipopolysaccharide (LPS; 10 μL mL^−1^) treatment for 24 hours. Distilled water served as the control, and all experiments were conducted in triplicate.

To assess hypolipidemic activity, HepG2 cells were cultured into 6-well culture plates in DMEM/high glucose at a density of 1 × 10^6^ cells per well and cultured 24 hours. After that, the cells were mimicked hyperlipidemic condition by incubating with 800 μM acid oleic within 4 hours. Subsequently, the cells were incubated with 100 μg mL^−1^ of AK1 or AK3 for another 24 hours. Distilled water was utilized as the control, and each experiment was repeated three times.

### 2.7. MTT assay

The viability of cells was evaluated by the MTT assay according to previous description.^17^ Briefly, HepG2 or RAW264.7 cells were inoculated at 0.5 × 10^5^ cells per well in 96-well plate and let to adhere for 24 h. Afterward, the cells were pretreated with AK1, AK3 (10, 50 and 100 μg mL^−1^) or dimethyl sulfoxide (DMSO; 0.01%) as control for 24 h and then exposed to 5 μL of MTT solution (5 mg mL^−1^) for another 4 h. The current media were then removed and 100 μL DMSO was added to dissolve the purple MTT-formazan crystals. The color of solution was read at a wavelength of 570 nm by a microplate reader (Thermo Fisher Scientific, Inc., Waltham, MA, USA). The cell survival rate was calculated by comparing absorbance of samples with that of the control. The experiment was repeated at least three times.

### 2.8. Flow cytometry analysis

The protective effect of AK1 and AK3 on HepG2 cells against damage induced by oxidative stress from H_2_O_2_ was evaluated using flow cytometry analysis, in accordance with the procedure outlined in Hien *et al.*^17^ The stained cells were identified utilizing the BD FACSMelody™ Cell Sorter (BD Biosciences, San Diego, CA, USA) and subsequently analyzed employing the BD FACSChorus software (BD Biosciences, San Diego, CA, USA).

### 2.9. Assay for NO inhibitory effect

The samples' capacity to inhibit NO production was assessed in RAW 264.7 cells activated by LPS. RAW264.7 cells were grown in 96-well culture plates using DMEM/high glucose, with a cell density of 2 × 10^5^ cells per well. After 24 hours, the cells were incubated with AK1 or AK3 at different concentrations for 2 hours followed LPS (10 μL mL^−1^) treatment for 24 hours. Distilled water and NG-methyl-l-arginine acetate (l-NMMA; Sigma) were used as a negative and positive control, respectively. The amount of NO in the culture medium was performed by using Griess Reagent System (Promega Cooperation, WI, USA), according to the manufacturer's instruction. The standard curve was established employing known concentrations of sodium nitrite, with the absorbance being assessed at 540 nm. The percentage of NO inhibition was calculated as follows:2
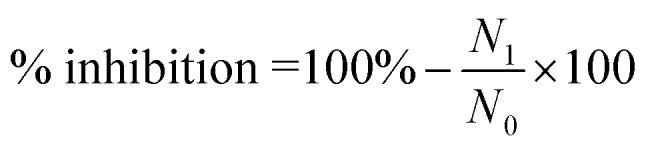
where *N*_1_ is the nitrite amount in the sample and *N*_0_ is the nitrite amount in the negative control.

The IC_50_ value was computed using GraphPad Prism 5 software (GraphPad Software, Inc., San Diego, California). Each experiment was repeated three times.

### 2.10. Lipid analysis

The extraction and assessment of intracellular lipids in HepG2 cells treated AK1 and AK3 were carried out using enzymatic techniques, following the procedure outlined in Hien *et al.*,^17^ and analyzed with an Olympus AU400 Clinical Chemistry Analyzer (Olympus Analyzers, Tokyo, Japan).

### 2.11. Real-time polymerase chain reaction (qPCR)

Total RNA was extracted from RAW 264.7 and HepG2 cells treated with AK1 or AK3 using TRIzol™ Reagent (Invitrogen, Singapore), following the manufacturer's instructions. The quantification of genes related to inflammation and lipid metabolism was conducted through quantitative real-time PCR, employing the Luna^®^ Universal One-Step RT-qPCR Kit (New England BioLabs Inc., UK) and the MyGo Pro real-time PCR instrument (IT-IS Life Science Ltd., Dublin, Republic of Ireland). The primer sequences are provided in Table S1.† Expression levels were determined using the 2^−ΔΔCt^ method and were normalized to β-actin, as detailed in Hien *et al.*^17^

### 2.12. Statistics

Significant distinctions between groups were evaluated through one-way ANOVA followed by a Tukey–Kramer posthoc analysis. Significance was established at *P* < 0.05.

## 3 Result and discussion

### 3.1. Preparation and characterization of astaxanthin/kaempferol co-encapsulated nanoparticles

The formation of astaxanthin/kaempferol nanoparticles was described in Fig. S1,† in which two commonly used methods were emulsion/solvent evaporation and spray drying. The success of the emulsification process in this study lies in the simultaneous use of two surfactants, lecithin and cremophor RH40. These surfactants stabilize the nanoastaxanthin/kaempferol by self-locating at the interface between the nanoparticles and water, with their hydrophilic head groups extending into the water and their hydrophobic tail groups interact with astaxanthin and kaempferol. Consequently, they reduce the interfacial tension between water and astaxanthin/kaempferol, forming a stable colloidal system. In addition, for the nanoparticle formulation, β-cyclodextrin was chosen as the carrier to facilitate the encapsulation of astaxanthin and kaempferol, thereby enhancing the stability of the nanoparticles. Previous studies have shown that β-cyclodextrin is one of the most commonly used cyclodextrins due to its compatibility with a wide range of bioactive compounds, which have molecular weights ranging from 200 to 800 g mol^−1^, and its relatively affordable cost.^20–22^ With a structure consisting of seven glucose subunits linked by α-(1,4) glycosidic bonds,^23,24^ the hydrophobic cavity of β-cyclodextrin interacts with the hydrophobic bioactive compounds, while the hydrophilic outer surface of β-cyclodextrin can improve their water solubility. The components used for nanoparticles fabrication, such as β-cyclodextrin, lecithin, and cremophor RH40, are non-toxic compounds and have been designated as safe by the Food and Drug Administration.^25–27^

**Fig. 1:**
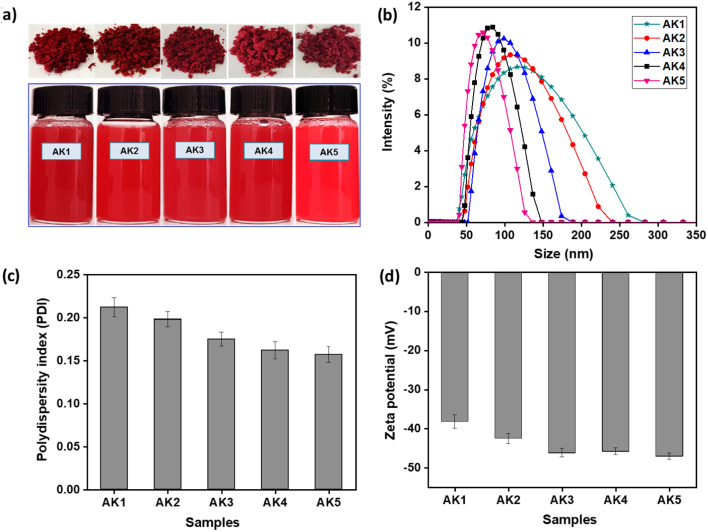
Characterization of astaxanthin/kaempferol co-encapsulated nanoparticles (AK). (a) Aqueous dispersion, (b) size distribution, (c) polydispersity index (PDI), and (d) zeta potential of AK with different loading contents of astaxanthin/kaempferol.

The strong compatibility of astaxanthin and kaempferol with surface-active agents such as lecithin and cremophor RH40, in conjunction with the carrier β-cyclodextrin, resulted in the formation of red-colored nanopowder with excellent water dispersibility (Fig. 1a). As illustrated in Fig. 1a, the nanoparticles dispersed very well in water, maintaining this state without aggregation even after several days of storage at room temperature. The hydrodynamic diameter, polydispersity index, and zeta potential of the astaxanthin/kaempferol nanoparticles were analyzed using dynamic light scattering (DLS). DLS analysis (Fig. 1b) indicated that the AK1 and AK2 nanoparticles, containing astaxanthin at 10 wt% and 9 wt%, respectively, had average diameters of 120 ± 3 nm and 109 ± 2 nm. These values were significantly smaller than the mean diameters reported by Oh *et al.*^28^ for astaxanthin-loaded lecithin nano-liposomes (ASTA@Lec NS), which ranged from 140 to 169 nm. Moreover, Fig. 1b demonstrated that the AK3 (7.5 wt% AST and 2.5 wt% KAE), AK4 (5 wt% AST and 5 wt% KAE), and AK5 (2.5 wt% AST and 7.5 wt% KAE) samples exhibited narrow dispersion in water, with average particle diameters of 97 ± 1, 83 ± 3, and 74 ± 4 nm, respectively. Previous reports described nanoastaxanthin with average particle diameters of approximately 200 nm.^29–31^ Therefore, the combination of astaxanthin and kaempferol in this study resulted in reduced particle sizes of the nanosystem to below 100 nm, even with a total content of these active compounds reaching 10%. Furthermore, the results indicated that the ratio of astaxanthin/kaempferol was proportional to the particle size of the fabricated nanoparticles.

The polydispersity index (PDI) is a measure of distribution that describes the width or spread of the size distribution of nanoparticle with ranges from 0.0 to 1.0. The PDI value of >0.5 indicates a wide size distribution and indicates that the sample contains large vesicles and aggregate; while the PDI value of <0.25 expresses a narrower distribution.^32^ The astaxanthin/kaempferol nanoparticles under the optimized formulation exhibited a narrow PDI (below 0.2), consistent with the results as shown in Fig. 1c. Specifically, the nanoparticles displayed good dispersion, with PDI values of 0.175 ± 0.008 for the AK3 nanoparticles, 0.162 ± 0.01 for the AK4 nanoparticles, and 0.157 ± 0.009 for the AK5 nanoparticles. In this study, the formation of astaxanthin/kaempferol nanoparticles was governed by the presence of surfactants and the encapsulating agent. Lecithin is an amphiphilic surfactant and highly miscible with astaxanthin, thus forming a hydrophobic mixture. Cremophor RH40 is a highly soluble aqueous emulsifier that solubilizes the astaxanthin/lecithin system in water. The synergy of surfactants (Cremophor RH40 and lecithin) along with the encapsulating agent (β-cyclodextrin) yielded well-defined astaxanthin/kaempferol nanoparticles exhibiting excellent water dispersion. Another important index, zeta potential, has a significant impact on nanoparticle stability. The greater the absolute value of zeta potential, which can result in increasing the stability of nanosamples. Particularly, dispersion with zeta potential values more than +20 or less than −20 mV is physically stable and beneficial for long-term storage.^33^ All astaxanthin/kaempferol nano-system showed a negative zeta potential, in the range of −38.2 to −47.1 mV (Fig. 1d), indicating that these nanosystems were highly physically stable.

**Fig. 2:**
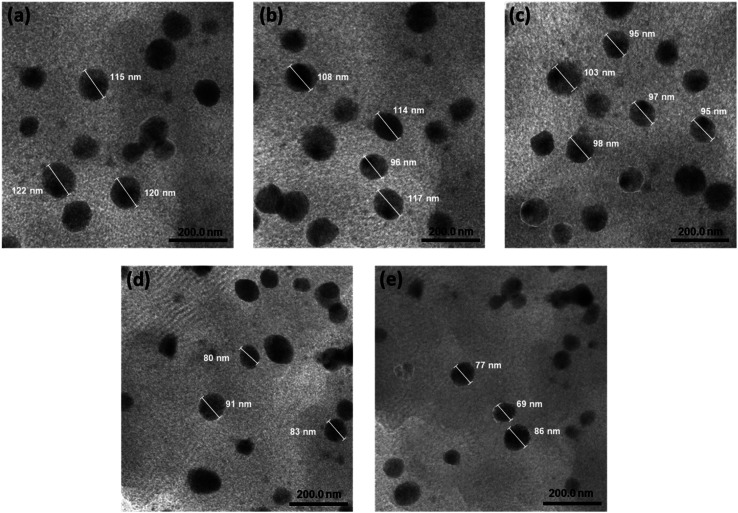
Transmission electron microscopy (TEM) image of AK1 (a), AK2 (b), AK3 (c), AK4 (d), and AK5 (e) samples (scale bar = 200 nm).

The morphology and mean diameter of the astaxanthin/kaempferol nanoparticles were also measured by transmission electron microscopy (TEM). As shown in Fig. 2a–e, these AK nanoparticles have a small spherical shape (around 100 nm) with little signs of aggregation. Among them, the AK3 sample (Fig. 2c) exhibited a small particle size and the most uniform distribution, which were in accordance with the data obtained by DLS.

### 3.2. Stability of nanoastaxanthin/kaempferol during storage

Astaxanthin is known for its poor stability, easily degrading when exposed to light and temperature variations during processing and storage. Some studies have shown that astaxanthin's stability can be improved through the use of antioxidants and nanoencapsulation techniques.^34–36^

**Fig. 3:**
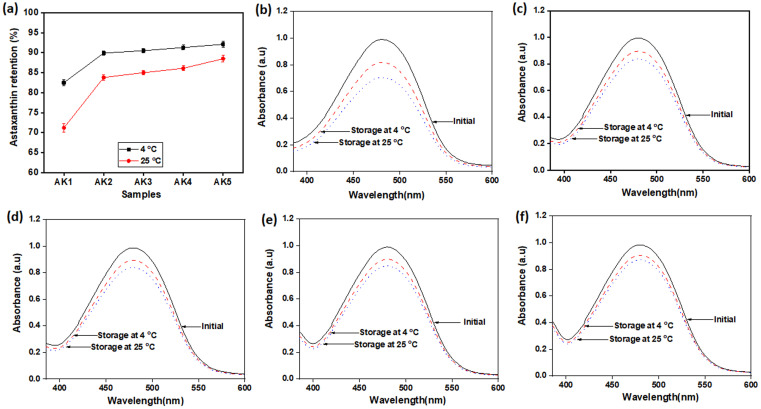
(a) Stability of astaxanthin/kaempferol co-encapsulated nanoparticles (AK), UV-vis spectra in THF solution of AK samples (b) AK1, (c) AK2, (d) AK3, (e) AK4, and (f) AK5 after a storage time of 6 months at different temperatures.

In this study, kaempferol played a dual role by acting synergistically with astaxanthin and by serving as an antioxidant to protect astaxanthin. The stability of nanoastaxanthin/kaempferol was evaluated after storage at room temperature and 4 °C for 6 months (Table 2 and Fig. 3). The astaxanthin/kaempferol nanopowder was filled into an amber glass bottle and flushed with nitrogen gas before airtight closure with a plastic cap. Under the ambient condition, astaxanthin was significantly degraded (Table 2 and Fig. 3). After 6 months of storage at room temperature, the remaining astaxanthin content in the AK1, AK2, AK3, AK4, and AK5 samples was 71.2 ± 1.1, 83.8 ± 0.7, 85.0 ± 0.5, 86.1 ± 0.6, and 88.5 ± 0.8%, respectively. There were no statistically significant differences in the astaxanthin content among the samples. However, the preparation of unstable carotenoids like astaxanthin into nano-powder form has been proven effective in enhancing the stability of the compound.^37,38^ These results indicate a promising application of nano-powder for preserving lipophilic bioactive compounds during storage. Additionally, we conducted further studies on the preservation of nano astaxanthin/kaempferol at 4 °C to improve the product's stability. Table 2 and Fig. 3 show that the remaining astaxanthin content in the samples after 6 months of storage at 4 °C increased, with values of 82.5 ± 0.8 (AK1 sample), 89.9 ± 0.6 (AK2 sample), 90.5 ± 0.5 (AK3 sample), 91.3 ± 0.6 (AK4 sample), and 92.1 ± 0.7% (AK5 sample).

**Table 2:** Stability of astaxanthin/kaempferol co-encapsulated nanoparticles after six months of storage at different temperatures

Storage conditions	Stability (retained astaxanthin content in astaxanthin/kaempferol co-encapsulated nanoparticles (%))				
	AK1	AK2	AK3	AK4	AK5
4 °C	82.5 ± 0.8	89.9 ± 0.6	90.5 ± 0.5	91.3 ± 0.6	92.1 ± 0.7
25 °C	71.2 ± 1.1	83.8 ± 0.7	85.0 ± 0.5	86.1 ± 0.6	88.5 ± 0.8

The degradation rate of astaxanthin at cold temperatures has decreased by approximately half compared to storage at room temperature. It is evident that good compatibility between astaxanthin and kaempferol, significantly enhances the stability of astaxanthin. Storing nanoastaxanthin/kaempferol products at lower temperatures can indeed improve the durability of astaxanthin, but it may not be suitable for production and practical applications. Therefore, a temperature of 4 °C is considered the ideal temperature for storing nanoastaxanthin/kaempferol samples.

According to Rizzo *et al.*,^13^ the combination of two antioxidants improved oxidative stress and anti-inflammatory parameters. In this study, the combination of astaxanthin and kaempferol not only increased the stability of the samples but also increased their antioxidant capacity when compared with astaxanthin alone (Table 3). The antioxidant activity of the nanoparticles was not proportional to the astaxanthin and kaempferol ratio. As shown in Table 3, the AK3 with the astaxanthin and kaempferol ratio of 7.5 and 2.5 reached the lowest IC_50_ value and the antioxidant activity of AK3 was the highest. Hence, AK3 was used for bioactivity evaluation and AK1 (nanoastaxanthin) was used to compare the effects of the combination of astaxanthin and kaempferol in the nanoparticle.

**Table 3:** Antioxidation activity of astaxanthin/kaempferol co-encapsulated nanoparticles

Samples	IC_50_ (μg mL^−1^)
AK1	2.82 ± 0.106
AK2	2.23 ± 0.132
AK3	1.32 ± 0.093
AK4	1.50 ± 0.062
AK5	1.88 ± 0.087
Ascorbic acid	19.43 ± 1.44

### 3.3. Cytotoxicity effect of astaxanthin/kaempferol co-encapsulated nanoparticles on HepG2 and RAW264.7 cells

**Fig. 4:**
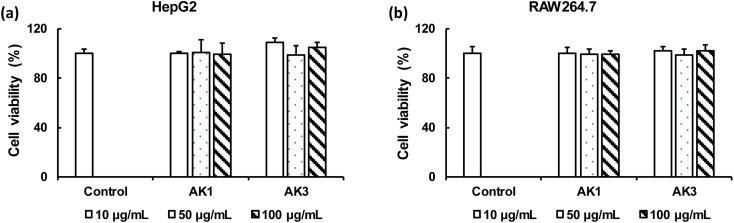
The MTT assay of HepG2 (a) and RAW264.7 (b) cells in the presence of AK1 and AK3 samples at varying concentrations for 24 h. Data are given as a mean ± SE (*n* = 3–5).

We conducted a comparative analysis of the toxicity impacts of AK1 and AK3 on HepG2 and RAW264.7 cells through an MTT assay. As illustrated in Fig. 4, the viability of HepG2 and RAW264.7 cells ranged from 98% to 108% when exposed to AK1 or AK3. These results indicate that neither AK1 nor AK3 induced noteworthy cytotoxic effects on HepG2 (Fig. 4a) or RAW264.7 (Fig. 4b) cells, even at the highest tested concentration of 100 μg mL^−1^.

### 3.4. Cytoprotective effect of astaxanthin/kaempferol co-encapsulated nanoparticles on HepG2 cells against oxidative stress-induced damage caused by H_2_O_2_

**Fig. 5:**
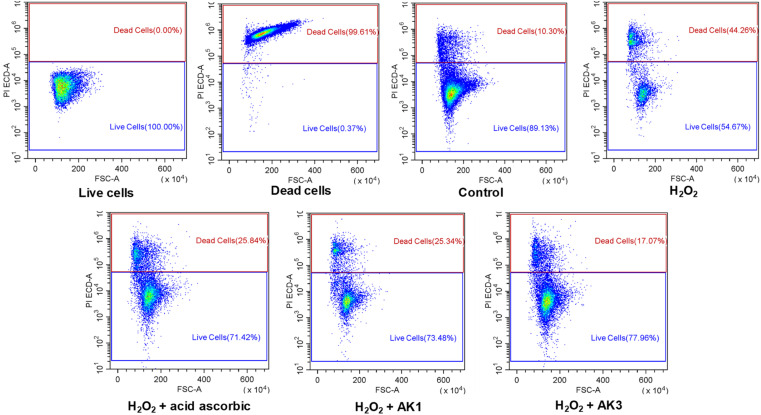
The astaxanthin/kaempferol co-encapsulated nanoparticles protected HepG2 cells against damages by oxidative stress induced by H_2_O_2_.

Numerous authors have reported that a combination of antioxidant has synergistic and complementary effects resulting in higher activities than those of each of the antioxidants on its own.^13–16^ In this study, we evaluated the cytoprotective effects of AK1 and AK3 against H_2_O_2_-induced cytotoxicity in HepG2 cells using flow cytometry analysis (Fig. 5).

As shown in Fig. 5, the cell viability was significantly decreased in cells treated with 5 mM H_2_O_2_. However, pretreatment of HepG2 with 10 μg mL^−1^ of AK1 or AK3 resulted in the recovery of cell viability to 73.48% and 77.96%, respectively. It was also indicated AK3-mediated cell recovery was more pronounced than AK1. Suggesting that combination of astaxanthin and kaempferol in nanoparticles has resulted in the improvement its potential protective effects against H_2_O_2_-induced cell mortality.

### 3.5. Inhibitory effect of astaxanthin/kaempferol co-encapsulated nanoparticles on NO production and mRNA level of iNOS, TNF-α, and IL-6

Nitric oxide (NO), a signaling molecule, involves in the regulation of diverse physiological and pathophysiological mechanisms in the immunological systems, cardiovascular, and nervous.^39^ One of the hallmarks of inflammation is the release of proinflammatory cytokines such as NO. We determined if AK1 or AK3 influences the production of NO by LPS stimulated RAW264.7 cells. Here, the cells accumulated NO in the culture medium when the cells exposed to LPS, and pretreated AK1 and AK3 inhibited NO production with an IC_50_ of ∼10 and 3.44 μg mL^−1^ (Table 3). Cells treated with combination of astaxanthin and kaempferol in nanoparticles had significantly more LPS stimulated NO production than that in astaxanthin alone in nanoparticles. The inhibitory effect on NO production of AK3 was 22.65%, 58.98%, 82.13%, and 89.88% at 0.08, 0.4, 2 and 10 μg mL^−1^, respectively (Table 4).

**Table 4:** The astaxanthin/kaempferol co-encapsulated nanoparticles inhibit NO in LPS stimulated RAW264.7 cells

Inhibition of NO production (%)			Inhibition of NO production (%)	
Concentration (μg mL^−1^)	AK1	AK3	Concentration (μg mL^−1^)	L-NMMA
0.08	0.66 ± 0.70	22.65 ± 1.05	0.8	9.57 ± 0.29
0.4	5.60 ± 1.40	58.98 ± 1.40	4	24.36 ± 0.72
2	12.03 ± 1.40	82.13 ± 0.70	20	80.73 ± 1.86
10	46.62 ± 2.09	89.88 ± 1.75	100	99.39 ± 2.59
IC_50_ (μg mL^−1^)	>10	3.44 ± 0.23	IC_50_ (μg mL^−1^)	7.92 ± 0.79

**Fig. 6:**
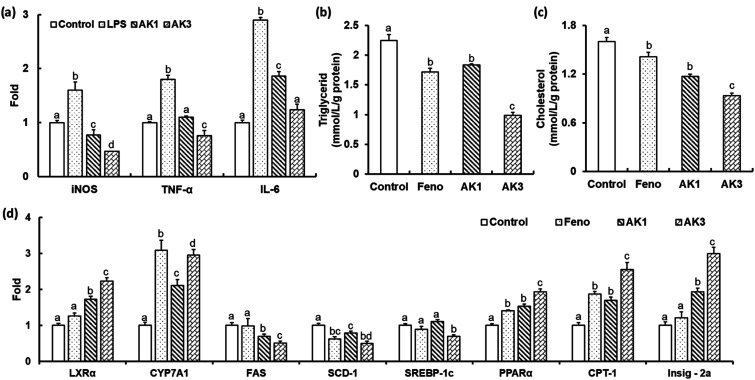
The effect of astaxanthin/kaempferol co-encapsulated nanoparticles on expression of genes related to inflammation in RAW264.7 (a), cellular lipid levels (b and c), and the expression of genes related to lipid metabolism (d) in HepG2 cells. The data were expressed in mean ± SEM (*n* = 3). Values not sharing a common superscript differ significantly at *P* ≤ 0.05.

Activated macrophages produce a large amount of NO *via* nitric oxide synthase (iNOS) and cyclooxygenase (COX), as well as the proinflammatory cytokines interleukin-6 (IL-6) and tumor necrosis factor-α (TNF-α).^40^ AK1 inhibited the gene expression of iNOS, TNF-α and IL-6 in LPL stimulated RAW264.7 cells (Fig. 6a). These effects were further enhanced by the combination of astaxanthin and keampferol. The LPS- stimulated gene expression of iNOS, TNF-α and IL-6 were significantly decreased by −70%, −58% and −57%, respectively, in AK3 treated RAW264.7 cells (Fig. 6a). These results suggest that AK1 and AK3 reduce the inflammatory response by suppressing the production of inflammatory cytokines and mediators. Anti-inflammatory activity is higher in the combination of astaxanthin and kaempferol than in astaxanthin alone.

### 3.6. Inhibitory effect of astaxanthin/kaempferol co-encapsulated nanoparticles on lipid accumulation by regulating genes participating in lipid metabolism in HepG2 cells

In both animal models and obese humans, NAFLD could be characterized by excessive accumulation of cellular lipids associated with inflammation, which can eventually progress to mitochondrial dysfunction, insulin resistance, and cellular injury.^41,42^ Our results showed that AK1 and AK3 exhibited effectively preventing of intracellular cholesterol and TG accumulation in HepG2 cells (Fig. 6b and c). Cells incubated with AK1 had a cholesterol- and TG-lowering effect of −18 and 27% respectively when compared with the control. The reduction in levels of cholesterol (−42% and −20%) and TG (−56% and −46%) in HepG2 cells incubated with AK3 was higher than that of control and AK1, respectively. AK3 exhibited a synergistic effect on dyslipidemia.

Cholesterol 7α-hydroxylase (CYP7A1), a crucial rate-limiting enzyme, holds a significant position in regulating the equilibrium between cholesterol and bile acid within the liver's bile acid biosynthesis pathway.^17^ Inhibition of *de novo* cholesterol production in the liver, coupled with an enhanced conversion of cholesterol into bile acids, results in reduced serum cholesterol levels. LXRα is a nuclear receptor that is involved in the regulation of cholesterol and bile acids metabolisms by regulating the transcription of CYP7A1.^17^ Our obtained results revealed that AK1 and AK3 could upregulate LXRα and CYP7A1 (Fig. 6d), suggesting that astaxanthin, either alone or in combination with KAE in nanoparticles, could facilitate the removal of excess cholesterol by promoting its conversion into bile acids within HepG2 cells.

Sterol regulatory element-binding protein 1 (SREBP-1c), along with its downstream genes stearoyl-CoA desaturase-1 (SCD1) and fatty acid synthase (FAS), plays a pivotal role in regulating the synthesis of free fatty acids (FFA) and triglycerides (TG).^17^ Carnitine palmitoyltransferase 1 (CPT-1), a critical rate-limiting enzyme, represents the initial step in facilitating the entry and β-oxidation of long-chain fatty acids into mitochondria.^17^ Peroxisome proliferator-activated receptor (PPARα), a nuclear receptor, exerts a crucial influence on lipid homeostasis by managing the equilibrium between fatty acid synthesis and oxidation.^17^ Notably, the expression of PPARα is notably reduced in cases of non-alcoholic fatty liver disease (NAFLD).^43^ In our current investigation, the results we obtained showed that HepG2 cells treated with AK1 and AK3 exhibited significant increases in the expression levels of PPARα and CTP-1, while the expression levels of FAS and SCD-1 were decreased (Fig. 6d). Moreover, it's important to note that AK3, but not AK1, significantly decreased the expression levels of SREBP-1c. As previously demonstrated in our earlier work,^43^ the inhibition of SREBP-1c expression is regulated by PPARα and/or Insig-2a. This regulation occurs through post-translational modification of SREBP1, initiated by the translocation of the precursor protein SREBP1, along with SCAP, from the endoplasmic reticulum (ER) to the golgi. This translocation is prompted by SREBP phosphorylation. The precursor protein SREBP1 (pSREBP1) is then cleaved by two separate proteases, S1P and S2P, resulting in the release of the mature form of SREBP1. Insig-2a enhances the presence of SREBP1 on the ER membrane, thereby inhibiting SREBP1 processing. In this study, we observed that HepG2 cells stimulated with either AK1 or AK3 exhibited a significant increase in the expression of the Insig-2a gene, with higher expression levels observed in the AK3-treated cells compared to the AK1-treated cells (Fig. 6d). Based on the aforementioned results, it is evident that the combination of astaxanthin and kaempferol in nanoparticles can substantially enhance cholesterol metabolism, modulate fatty acid synthesis and oxidation, thereby effectively preventing NAFLD.

## 4 Conclusions

In this research, astaxanthin/kaempferol co-encapsulated nanoparticles were successfully fabricated and showed good dispersibility in water, a small average particle size, and high stability. Among them, nanoparticles containing 7.5 wt% AST and 2.5 wt% KAE exhibited the best physicochemical properties and biological activities. These nanoparticles displayed a spherical shape with an average particle diameter of 97 nm, a narrow polydispersity (PDI = 0.175), and a negative surface potential (*ζ* = −46.2 mV). Moreover, the astaxanthin content in the nanoastaxanthin/kaempferol formulation remains consistently above 90% after 6 months of storage at 4 °C. Furthermore, these nanoparticles were nontoxic, even at the highest tested concentration of 100 μg mL^−1^ on HepG2 or RAW264.7 cells. Our research also substantiates that this nano-formulation enhances the antioxidant, anti-inflammatory, and lipid-lowering activities of nanoastaxanthin/kaempferol across various cell lines. Finally, the astaxanthin/kaempferol nanoparticles exhibited potential value in safe and efficient application of astaxanthin and kaempferol.

## Author contributions

Conceptualization, Mai Ha Hoang, Hoang Thi Minh Hien; data analysis, Hoang Thi Minh Hien, Ho Thi Oanh; methodology, Ho Thi Oanh, Mai Ha Hoang, Hoang Thi Minh Hien; project administration, Hoang Thi Minh Hien; validation, Ngo Thi Hoai Thu, Nguyen Van Hanh, Hoang Thi Minh Hien, and Ho Thi Oanh; writing – original draft, Hoang Thi Minh Hien, Ho Thi Oanh; writing – review & editing, Hoang Thi Minh Hien, Ho Thi Oanh, and Mai Ha Hoang. All authors have read and agreed to the published version of the manuscript.

## Conflicts of interest

The authors declare that they have no conflict of interest.

## Supplementary Material

RA-013-D3RA06537E-s001
