# Supplementary material for: Co-encapsulated astaxanthin and kaempferol nanoparticles: fabrication, characterization, and their potential synergistic effects on treating non-alcoholic fatty liver disease
Source: RSC Adv. 2023 Dec 1;13(50):35127–36. doi: 10.1039/d3ra06537e (PMC10691322; doi:10.1039/d3ra06537e)
Supplement: RA-013-D3RA06537E-s001 [file RA-013-D3RA06537E-s001.pdf]

Electronic Supplementary Material (ESI) for RSC Advances.
This journal is © The Royal Society of Chemistry 2023

Supplementary material

Co-encapsulated astaxanthin and kaempferol nanoparticles: Fabrication, characterization, and their potential synergistic effects on treating non-alcoholic fatty liver disease

Ho Thi Oanh,[a] Ngo Thi Hoai Thu,[b] Nguyen Van Hanh,[b] Mai Ha Hoang,[*a,c] and Hoang Thi Minh Hien[*b,c]

[a.] *Institute of Chemistry, Vietnam Academy of Science and Technology, 18 Hoang Quoc Viet street, Cau Giay, Ha Noi, Vietnam*
[b.] *Institute of Biotechnology, Vietnam Academy of Science and Technology, 18 Hoang Quoc Viet street, Cau Giay, Ha Noi, Vietnam.*
[c.] *Graduate University of Science and Technology, Vietnam Academy of Science and Technology, 18 Hoang Quoc Viet Street, Cau Giay, Ha Noi, Vietnam*

Supplementary Figure

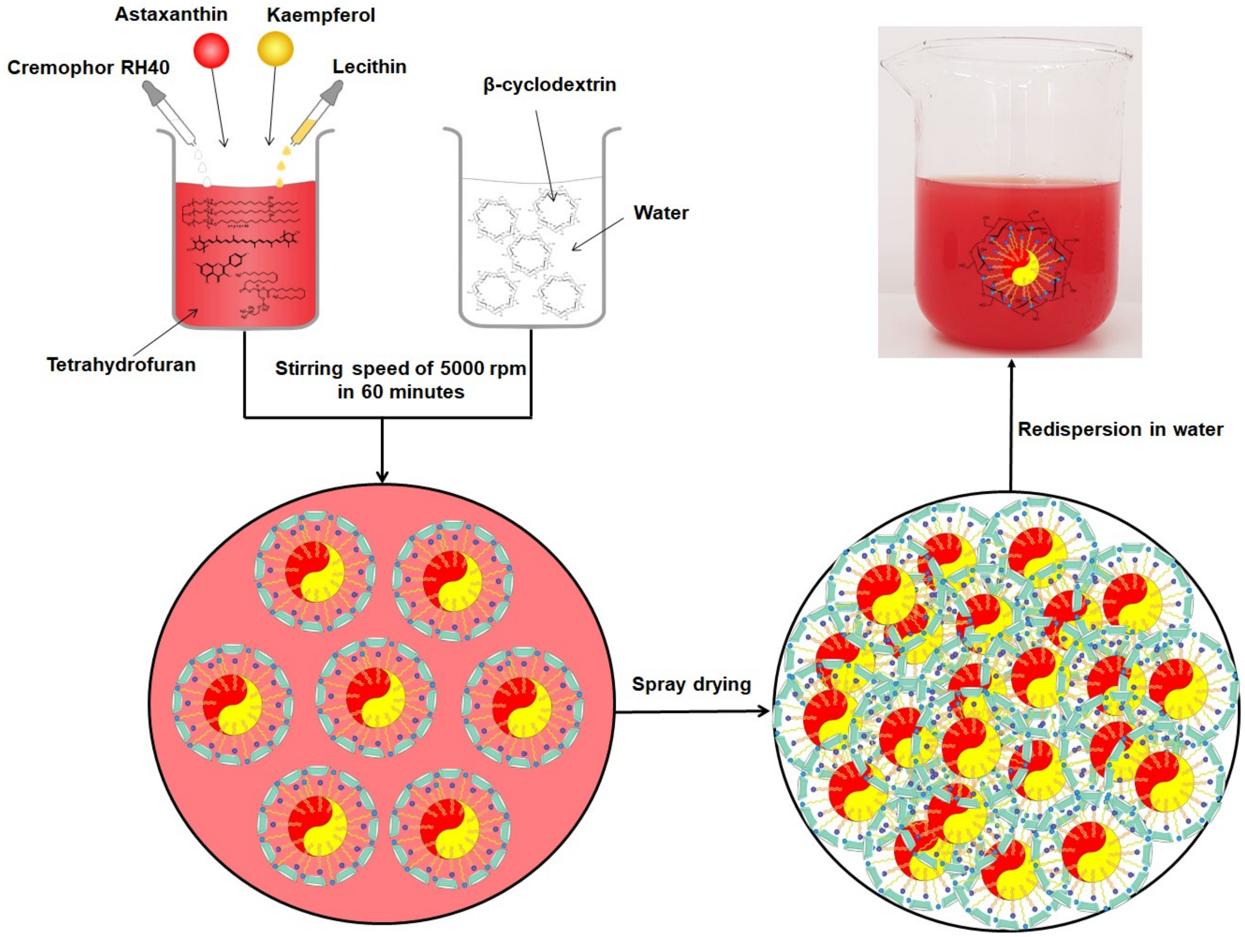

Fig. S1 Schematic illustration of astaxanthin/kaempferol nanoparticles formation

Supplementary Table

Table S1. Primers used for qPCR.

Gene	Primer (5´-3´)	Sequence
miNOS	Forward	AGAAGGAAATGGCTGCAGAA
	Reverse	GCTCGGCTTCCAGTATTGAG
mTNF-α	Forward	CCCAGATAGATGGGCTCAT
	Reverse	CAGGGACCTCTCTCTAATCA
mIL-6	Forward	TGAACTCCTTCTCCACAAGCG
	Reverse	GCTCTTCTGGGAAGTAGACAG
mGAPDH	Forward	CAACGAATTTGGCTACAGCAAC
	Reverse	ACTGTGAGGAGGGGAGATTCAG
hβ-actin	Forward	TGGCACCCAGCACAATGAA
	Reverse	CTAAGTCATAGTCCGCCTAGAAGCA
hLXRα	Forward	CGGGCTTCCACTACAATGTT
	Reverse	CTTCTCGATCATGCCCAGTT
hCYP7A1	Forward	AACACCATTCCAGCGACTTTCT
	Reverse	GATTGCCTTCCAAGCTGACTTT
hFAS	Forward	AGCTGCCAGAGTCGGAGAACTTG
	Reverse	CAAGAACTGCACGGAGGTGTTGG
hSCD-1	Forward	TGGGTGGCTGCTTGTG
	Reverse	GCGTGGGCATGAAG
hCTP-1	Forward	TGAGACCAAACAAAGTGATGATGTCAG
	Reverse	AGACGGTGGAACAGAGGCTGAAG
hSREBP-1c	Forward	CGGAGCCATGGATTGCACTTT

	Reverse	GATGCTCAGTGGCACTGACTCTTC
hInsig-2a	Forward	CGTTCTTGGTTACCATGTA
	Reverse	TGATGAGATTTTTCTGCGATAACTTT
hPPARα	Forward	ACGGAAAGCCCACTCTGCCCCCTCTC
	Reverse	CTTGTCCCCGCAGATTCTACATTCG

(m), indicates mouse genes (h) indicates human genes;
